# The Longitudinal Effect of Psychological Distress on Internet Addiction Symptoms Among Chinese College Students: Cross-Lagged Panel Network Analysis

**DOI:** 10.2196/70680

**Published:** 2025-05-02

**Authors:** Yuxuan Jiang, Chuman Xiao, Xiang Wang, Dongling Yuan, Qian Liu, Yan Han, Jie Fan, Xiongzhao Zhu

**Affiliations:** 1 Medical Psychological Center Second Xiangya Hospital of Central South University Changsha China; 2 Central South University Medical Psychological Institute of Central South University Changsha China; 3 National Clinical Research Center on Mental Disorders (Xiangya) Changsha China; 4 National Center for Mental Disorder Changsha China

**Keywords:** internet addiction, depression, anxiety, stress, cross-lagged panel network analysis, network analysis, college students, psychological distress

## Abstract

**Background:**

There is a growing amount of evidence suggesting high rates of co-occurring internet addiction (IA) symptoms and psychological distress in youth. However, the extent to which IA symptoms develop over time, how they interact with psychological distress symptoms dynamically, and how they predict one another remain unclear. Additionally, what specific types of distress, including depression, anxiety, and stress, are more closely associated with IA symptoms remains inconclusive.

**Objective:**

This longitudinal study aimed to explore the development of and changes in IA symptoms over time and the directional relationship between IA and various psychological distress symptoms.

**Methods:**

This study followed a sample of 2497 Chinese college students (mean age 19.14, SD 0.72 years) across 3 waves of a data collection span of 2 years. Their IA and psychological distress symptoms were assessed at baseline (T1), 12-month follow-up (T2), and 24-month follow-up (T3). We used network analysis to examine the network structure of IA symptoms at each wave and cross-lagged panel network (CLPN) analysis to investigate longitudinal associations between IA symptoms and psychological distress, including depressive, anxiety, and stress symptoms.

**Results:**

The cross-sectional networks of IA symptoms at 3 time points showed high similarity in terms of structure, existence of edges, and centrality indices. Nodes A2 (excessive use), A1 (salience), and A5 (lack of control) emerged as nodes with the highest expected influence (EI) centrality in the IA symptom networks across time (A2: EI=1.13 at T1, 1.15 at T2, 1.17 at T3; A1: EI=1.10 at T1, 1.13 at T2, 1.15 at T3; A5: EI=0.86 at T1, 0.88 at T2, 0.92 at T3). CLPN analysis revealed that psychological distress predicts IA symptoms but not the other way around. Depressive symptoms played a key role in predicting various IA-related problems (T1 to T2, edge weight=0.11; T2 to T3, edge weight=0.28; T1 to T3, edge weight=0.22) and served as bridge symptoms connecting IA and psychological distress (T1 to T2: bridge–expected influence [BEI]=0.15; T2 to T3: BEI=0.14; T1 to T3: BEI=0.19).

**Conclusions:**

Findings revealed a relatively stable network structure of IA symptoms among college students and suggested that psychological distress, especially depressive symptoms, may play a central role in activating IA symptoms over time. These results provide evidence for understanding the directional relationship between the central characteristics of distress symptoms and IA. The study also underscores the importance of depressive symptoms in their co-occurrence with IA, indicating that the key and bridge symptoms identified in this study can be prioritized as targets for preventing and treating IA in Chinese youth. Through identification and early intervention of depressive symptoms, we may avoid the progression of co-occurring issues, leading to more effective treatment outcomes.

## Introduction

The internet has brought numerous benefits to young generations, such as facilitating online communication and providing easy access to educational resources [1]. However, it has also given rise to a growing concern in public health worldwide—internet addiction (IA) [2]. IA refers to “excessive or poorly controlled preoccupations, urges or behaviors regarding computer use and Internet access that lead to impairment or distress” [3]. College students, in particular, have become a focal point within the broader internet user population. The pooled prevalence of IA is estimated to be around 11% among college students in China [4-6]. Given the high prevalence, negative impact, and changing nature of IA in college students, it is crucial to enhance our understanding of IA in this user group in order to develop effective early intervention strategies [7].

College students experience a critical developmental period marked by drastic physical, psychological, and biological changes [8], Additionally, as students transition to college, their living environment undergoes significant changes. With more free time, less supervision, and easier access to the internet, many students may become more prone to excessive online time. These environmental and psychological shifts increase the risk of IA among college students [9]. Many previous studies have investigated the prevalence, persistence, and correlates of IA in college students [4,10,11]. However, problematic internet use is a dynamic process with fluctuating patterns over time. More research is needed to investigate the robust developmental characteristics of IA. Therefore, a long-term study investigating these patterns may provide clearer insights into the development and trajectory of IA symptoms in this population.

It is also worth noticing that IA symptoms are rarely an isolated issue. To date, a significant body of studies has found the co-occurrence or correlation of IA with various types of psychological distress, including stress, depression, and anxiety [12-15]. However, 2 key questions remain regarding the relationship between psychological distress and IA symptoms. First, the directional relationship between IA symptoms and psychological distress is not fully understood. Various studies and theory models have yielded inconsistent conclusions regarding the directional relationship between IA symptoms and psychological distress. The cognitive behavioral model of pathological internet use posits that negative emotions lead to maladaptive cognitions for internet use, thereby resulting in IA [16]. The theory of compensatory internet use suggests that individuals with depressive symptoms tend to develop certain internet use motivations, such as escaping reality through internet use to alleviate their depressive symptoms [17]. The interaction of person-affect-cognition-execution (I-PACE) model also suggests that individuals with negative emotions are more likely to experience negative emotional or cognitive reactions (eg, a craving to regulate mood through addictive substances) when faced with tempting or stressful situations. This can lead to IA [18]. Despite their divergent foundations in maladaptive cognitions, psychological resource theory, and neurobiological processes, all 3 models converge in identifying negative emotions as a predisposing factor for IA. Many empirical studies have provided support for these theories, as they have found that individuals with psychological distress are more likely to develop IA [19-22].

However, some research suggests that addictive disorders might precipitate other psychiatric disorders [23]. Specifically, individuals with addictive behaviors tend to spend more time and energy on the internet and experience frustration or depression when returning to real-life demands [24,25]. Studies have also proposed a bidirectional relationship, where IA and other psychiatric disorders may mutually increase susceptibility [26,27]. Yet, the existing data supporting these hypotheses remain inconclusive.

Whether IA symptoms trigger psychiatric distress or vice versa is still unclear. Existing research has several limitations. First, most studies have relatively short follow-up periods, which makes it challenging to capture the full scope of changes and instability in IA symptoms and their co-occurrence with negative emotional symptoms. Thus, further studies with longer tracking periods are needed to test these questions more comprehensively. Second, previous research has mostly relied on latent variable models [28,29], which are limited in their ability to explore IA symptoms and their relationship with specific types of distress at the item level. In other words, the specific associations between different forms of psychological distress and IA remain unclear. Some studies suggest that depressive symptoms may have a stronger correlation with IA compared to stress and anxiety [12,30], while other researchers have found a stronger correlation between anxiety and IA [30]. Further investigation into how different aspects of distress specifically interact with IA symptoms is needed, as this could help identify precise risk factors and inform targeted interventions for co-occurring issues, which are crucial for improving health outcomes among college students.

Related studies have provided limited insight into the changes in IA symptoms, and their relationship with different types of distress may be related to the restriction of the method used. Network analysis, based on the network theory of mental disorders, allows for the visualization of different symptoms within a network, helping assess their complex relationships and interactions at the symptom level [31,32]. In addition to constructing cross-sectional networks, cross-lagged panel network (CLPN) analysis is commonly used with multiwave longitudinal data to explore the dynamics within and between psychological constructs over time. CLPN analysis helps identify the most central symptoms in terms of their predictive relationships, revealing which symptoms influence others or are influenced by them. Given these strengths, this study used network analysis as the primary statistical approach.

To sum up, this longitudinal study aimed to explore (1) the development and changes of IA symptoms over time, (2) the directional relationship between IA and psychological distress, and (3) the distinct effects of various aspects of psychological distress on IA symptoms. To address these questions, 3 cross-sectional IA symptom networks and 3 CLPN models were constructed. The findings of this study could provide valuable insights into the progression of IA symptoms and the specific patterns of interaction between IA and psychological distress, helping identify key targets for intervention strategies.

## Methods

### Participants and Procedures

Participants in this study were recruited from a university in Hunan Province, China. Three waves of surveys were conducted between 2019 and 2021. During the 2019-2020 academic year, all 3800 freshmen were invited to participate in a baseline survey conducted in December 2019. Survey details were provided in the questionnaire, which were distributed among the students during a class break.

Ultimately, 3578 (94.2%) freshmen agreed to participate and completed the questionnaires at baseline (T1; October 2019). In 2020, due to the rapid spread of COVID-19, the university was closed, and all students were placed in quarantine to protect public health. Consequently, an online survey was conducted through the Questionnaire Star platform for the second wave of data collection in June 2020, with 3205 (89.6%) students completing the survey. The third wave of data collection took place in December 2020, after the epidemic had been better controlled, and on-site surveys resumed. A total of 2953 (92.1%) students completed the third-wave survey. Missing data were handled using multiple imputations with the *mice* package in R (R Foundation for Statistical Computing) [33]. Ultimately, 2497 (84.6%) students who completed the questionnaires across all 3 waves were included in the final analysis. At each time point (baseline, T1; 12-month follow-up, T2; 24-month follow-up, T3), self-reported questionnaires covering demographics, psychological distress, and IA were administered during a class break.

### Ethical Considerations

The study was approved by the Ethics Committee of the Second Xiangya Hospital of Central South University (approval number: 81671341). All participants were informed about the study’s purpose and procedures. Written informed consent was obtained from all participants before they completed the questionnaires. The data underwent de-identification processing.

### Psychological Assessments

#### Internet Addiction

IA symptoms were measured using Young’s Internet Addiction Test (IAT) [34]. The IAT includes 20 items rated on a 5-point Likert scale, ranging from 1 (rarely) to 5 (always), with the total score ranging from 0 to 100. Higher scores indicate a greater level of IA. The Chinese version of the IAT has been previously validated [35,36]. In this study, Cronbach α was .87 for T1, .89 for T2, and .90 for T3. Confirmatory factor analyses were performed to confirm the construct validity of the IAT. The 1-factor model and the classical 6-factor model (salience, excessive use, neglect work, anticipation, lack of control, neglect social life) proposed by Widyanto et al [37] were both evaluated. The internal consistencies of the 6 factors of the IAT are shown in Table S1 in Multimedia Appendix 1. The models demonstrated a good fit at all time points, as indicated in Table S2 in Multimedia Appendix 1.

#### Psychological Distress

The distress experiences were measured using the Depression Anxiety Stress Scale (DASS), which consists of 21 items divided into 3 subscales, with 7 items each, measuring depression, anxiety, and stress [38]. Items were rated on a 4-point Likert-type scale, ranging from 0 (did not apply) to 3 (applied most of the time). Total scores for each dimension were obtained by summing the item scores of the subscales, with possible totals ranging from 0 to 21 for each of the 3 factors. Cronbach α was .93 for T1 and .95 for both T2 and T3.

### Statistical Analysis

The mean (SD) scores for the IAT and DASS across the 3 waves were calculated using IBM SPSS Statistics (version 27). Network analysis was conducted using R Studio (version 4.2), encompassing network estimation, network comparison, and CLPNs. As mentioned before, missing data were addressed through multiple imputations using the *mice* package in R [33].

#### Network Estimation

To estimate IA network structures at each wave, the *EBICglasso* function from the *qgraph* package (version 1.5) was used. Once network models were estimated for each time point, node centrality and edge weights were assessed. Centrality was measured by the index of the expected influence (EI), which quantified how strongly and directly a symptom node was associated with all other nodes in the network. Edge weights were numerical values assigned to edges representing regularized partial correlations in networks.

#### Accuracy, Stability, and Reproducibility of IA Networks

The accuracy, stability, and reproducibility of the IA network structures were assessed using the *bootnet* package (version 1.1.0) with a bootstrapping procedure with 1200 iterations. First, the accuracy of the edge through the 95% CI of the bootstrapped edge weight was estimated. When the bootstrapped CIs were wide, it became hard to ensure the stability of an edge. Second, the stability of the node centrality was tested using subset bootstrapping. The centrality stability (CS) coefficient was adopted as a reference index. For the CS coefficient, values greater than or equal to 0.25 indicated acceptable stability, while values greater than or equal to 0.5 indicated good stability. In addition, edge weight difference tests and centrality difference tests were conducted to assess whether differences between edge weights or node centralities were statistically significant. Lastly, to estimate the power, the *netSimulator* function was used to simulate data under a given network model and expected network structure. Epskamp et al [32] proposed 3 properties (sensitivity, specificity, and correlation between edge weights of the true and estimated networks) in the exploration of an appropriate sample size to detect true effect sizes. The 3 properties should be high in the appropriate sample. Results of all the edge weight accuracy, correlation stability, bootstrapped difference tests, and power analysis can be found in Multimedia Appendix 1.

#### Network Comparison

The network comparison test in the *NetworkComparisonTest* (NCT) package in R was used to examine the IA network structure difference over time through a permutation test [39]. Network invariance, global strength, and edge weights were calculated to describe differences in the global and local characteristics of IA networks.

#### Cross-Lagged Panel Networks

To examine prospective associations between IA symptoms and psychological distress, 3 CLPNs (T1→T2, T2→T3, T1→T3) were computed. In the networks, the 21 items of DASS were used as individual nodes representing psychological distress symptoms and the 6 subdimensions of Young’s IAT were used as nodes representing IA symptoms. This factor-item approach reduced data dimensionality, while preserving crucial subdimension information. It also ensured a balanced number of IA symptoms and distress nodes, meeting the node equilibrium requirements for network analysis [32].

The *glmnet* package [40] and the *qgraph* package were used to calculate regressions and plot all graphs of the CLPNs, respectively. In the CLPNs, variable contributions were represented as both autoregressive effects and cross-lagged effects, which were quantified by a series of regularized regressions and the penalized maximum likelihood with the LASSO penalty. We set the tuning parameter (λ=0.5) to increase the sparsity of a graph and reduce the possibility of overfitting problems. This calculation allowed for the estimation of CLPN centralities for in-prediction, out-prediction, and bridge items, thereby clarifying the directed relationships between IA and distress. Specifically, in-prediction was estimated by index of in–expected influence (IEI), which reflects the proportion of variance in a node at T2 explained by variables at T1. Out-prediction was estimated by out–expected influence (OEI), which reflects the influence of a node at T1 on variables at T2. In this study, we focused on cross-construct network centralities, excluding paths within the same construct, to better clarify the longitudinal interactions between IA and psychological distress. Additionally, 1-step and 2-step bridge–expected influence (BEI1 and BEI2) were calculated to identify bridge items linking IA symptoms with psychological distress.

#### Post hoc Stability and Accuracy Analysis of CLPNs

The accuracy and stability of the CLPNs was also estimated using 2 bootstrapping approaches implemented using the *bootnet* package in R. First, we calculated 95% CIs for edge weight accuracy via nonparametric bootstrapping (1200 bootstraps) to estimate network stability [32]. Second, the rank-order stability of centrality indices and edges were examined by computing the correlation stability coefficient [32].

## Results

### Descriptive Analysis

An independent-sample *t* test revealed no significant differences in study variables (IAT and DASS) between participants who completed all 3 waves (*t*=1.87, *P*=.06) and those who dropped out (*t*=1.52, *P*=.13), indicating that data were missing at random. The final sample included 2497 participants who completed assessments across all 3 waves, comprising 727 (29.1%) male and 1768 (70.8%) female participants, with ages ranging from 16 to 22 years (mean 19.14, SD 0.72) and education ranging from 11 to 15 years (mean 12.34, SD 0.71). Of the participants, 1066 (42.7%) grew up in urban areas and 1308 (52.4%) grew up in rural areas before age 12 years. Table 1 presents item descriptions, node labels, and the means (SDs) of psychological distress and IA symptoms.

**Table 1:** Item labels and descriptive statistics (N=2497).

Instrument and items		Node label	Baseline (T1)	12-month Follow-up (T2)	24-month Follow-up (T3)
			Mean (SD)	Mean (SD)	Mean (SD)
IAT^a^			44.92 (13.96)	43.35 (14.75)	43.86 (15.12)
	Salience	A1	11.42 (4.08)	10.96 (4.13)	11.04 (4.22)
	Excessive use	A2	10.87 (3.78)	10.64 (3.89)	10.85 (3.99)
	Neglect work	A3	7.26 (2.69)	6.85 (2.79)	6.87 (2.79)
	Anticipation	A4	4.28 (1.83)	4.32 (1.84)	4.47 (1.93)
	Lack of control	A5	7.19 (2.99)	6.66 (2.87)	6.71 (2.86)
	Neglect social life	A6	3.94 (1.78)	3.97 (1.68)	3.94 (1.69)
DASS^b^			11.27 (9.49)	9.00 (9.44)	9.13 (9.80)

^a^IAT: Internet Addiction Test.

^b^DASS: Depression Anxiety Stress Scale.

### Dynamic Changes in IA Networks

#### Network of IA Symptoms

**Figure 1:**
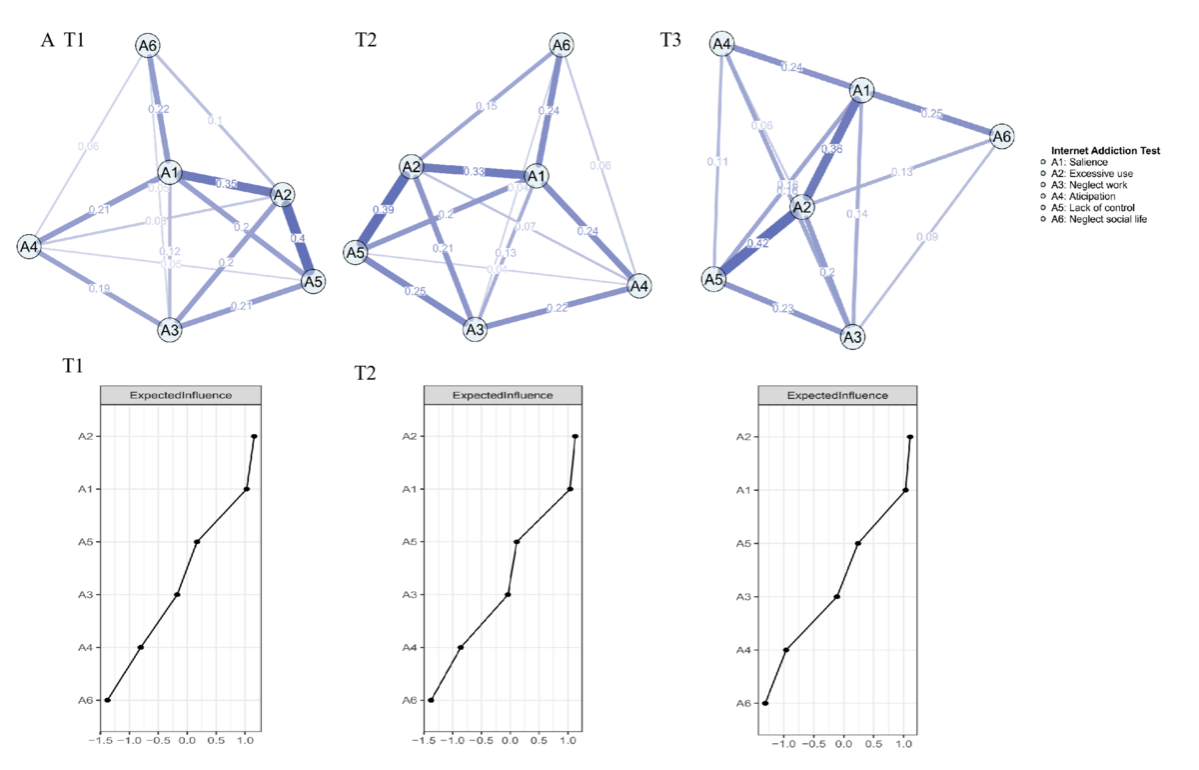
IA symptom networks of 3 waves of surveys. IA: internet addiction.

Figure 1 presents the IA symptom networks and centrality plots for T1, T2, and T3. The symptom networks across these 3 time points were similar. Notably, the edge between A2 (excessive use) and A5 (lack of control) consistently showed the highest weight across all 3 networks. Nodes A2 (excessive use), A1 (salience), and A5 (lack of control) demonstrated the highest EI values at each time point (T1: A2 EI=1.13, A1 EI=1.10, A5 EI=0.86; T2: A2 EI=1.15, A1 EI=1.13, A5 EI=0.88; T3: A2 EI=1.17, A1 EI=1.15, A5 EI=0.92). The results of the bootstrapped difference tests for edges and EI are presented in Figure S1 in Multimedia Appendix 1.

#### Network Comparison of IA Networks

We compared the edge strength, global strength, and network invariance between the networks at T1 and T2, as well as at T2 and T3. The results indicated no significant differences in edge strength between T1 and T2 (*P*=.17) or between T2 and T3 (*P*=.15). However, a significant global difference was observed between T1 and T2 (global strength T1/T2=2.44/2.57, S=0.13, *P*=.01) but not between T2 and T3 (global strength T2/T3=2.57/2.56, S=0.01, *P*=.69). Additionally, no significant differences in network invariance were found between T1 and T2 (M=0.05, *P*=.64) or between T2 and T3 (M= 0.06, *P*=.39).

### Potential Effect of Psychological Distress on IA Symptoms: CLPNs

**Figure 2:**
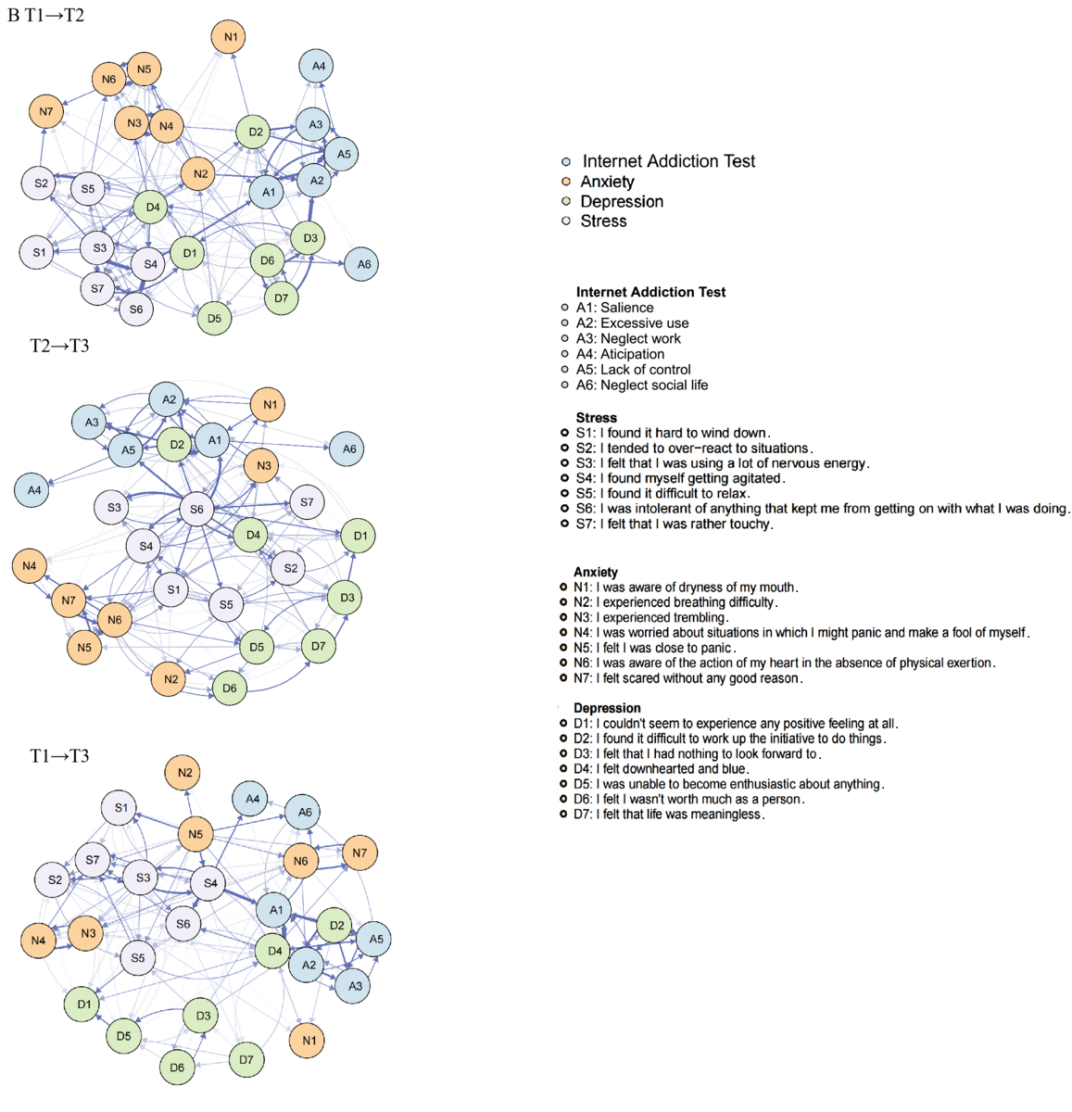
CLPNs established to analyze longitudinal associations between psychological distress and IA symptoms across shorter and longer time cycles. Arrows represent longitudinal relationships. CLPN: cross-lagged panel network; IA: internet addiction.

To further analyze the longitudinal associations between psychological distress and IA symptoms across shorter and longer time cycles, we established 3 CLPNs for T1 to T2, T2 to T3, and T1 to T3 (Figure 2). To enhance the visual interpretability of cross-lagged edges, we excluded autoregressive edges, as the plotting algorithm scales path thickness relative to the strongest path. After achieving regularization convergence and omitting the autoregressive paths, we identified 186 nonzero cross-lagged edges in the T1-to-T2 network, 195 in the T2-to-T3 network, and 160 in the T1-to-T3 network. A plot that includes these weaker edges can be found in Figure S2 in Multimedia Appendix 1.

**Figure 3:**
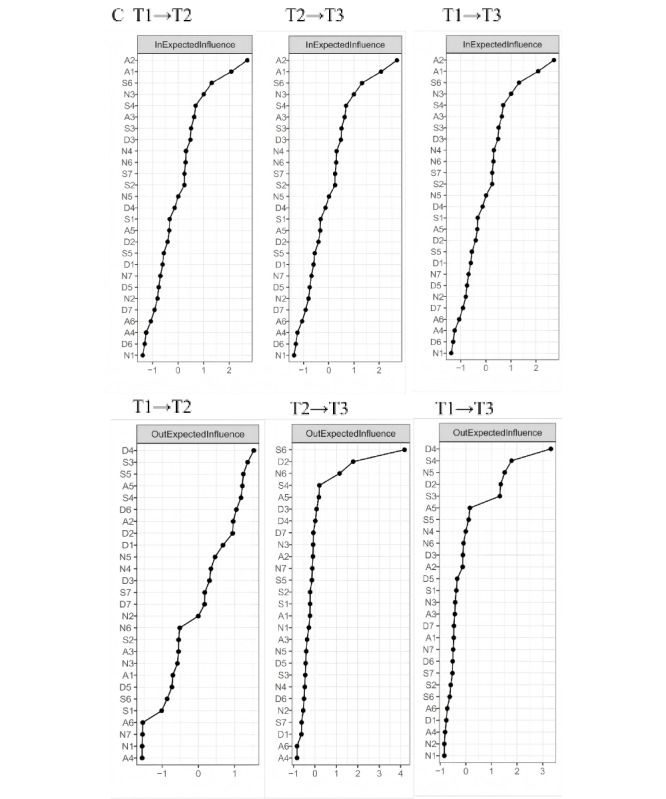
Centrality plots of CPNs. CPN: cross-lagged panel network.

**Figure 4:**
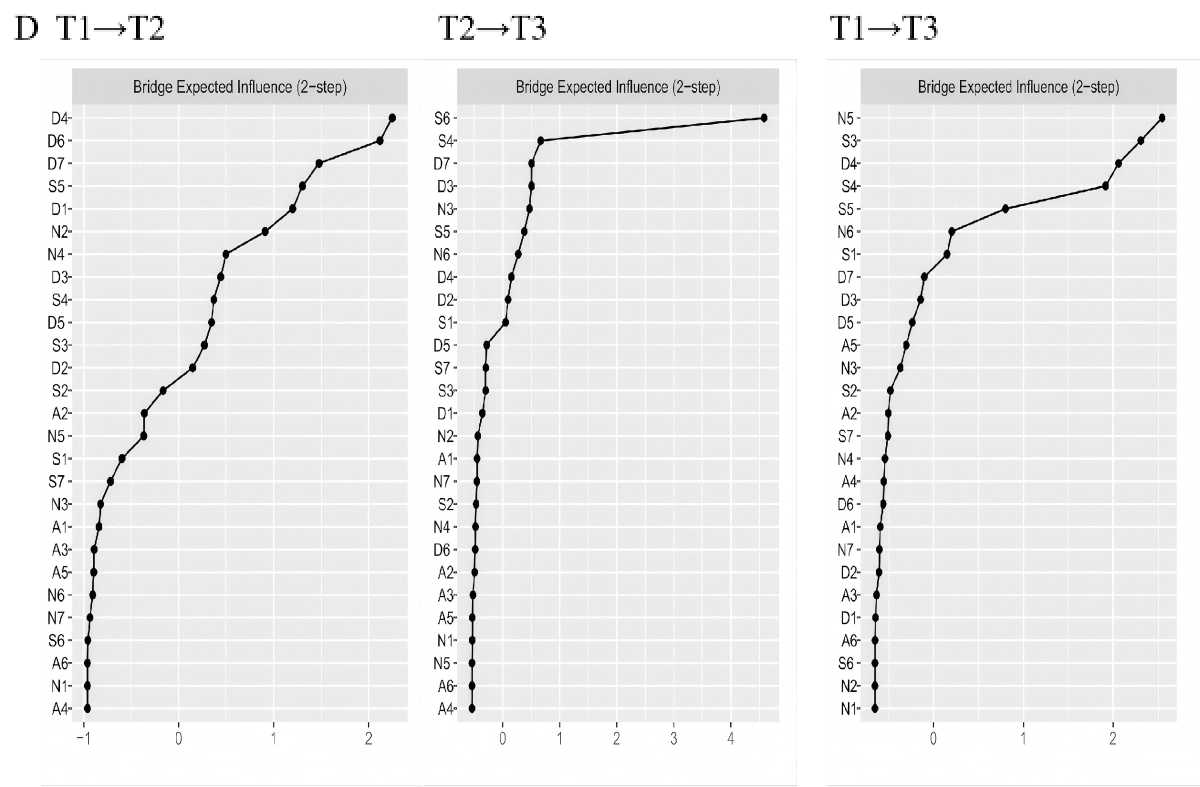
BEI (2-step) plots of CLPNs. BEI: bridge–expected influence; CLPN: cross-lagged panel network.

In the CLPNs for T1 to T2 (Figure 2), the strongest cross-lagged edge reflecting the longitudinal processes within IA symptoms was from A5 to A2 (“lack of control” to “excessive use”; edge weight=0.14). Within psychological distress symptoms, the strongest edge was from S4 to S6 (“I found myself getting agitated” to “I was intolerant of anything that kept me from getting on with what I was doing”; edge weight=0.14). When examining the longitudinal effects between IA and psychological distress, the most significant cross-lagged edges were from D3 to A2 (“I felt that I had nothing to look forward to” to “excessive use”; edge weight=0.11) and from D2 to A3 (“I found it difficult to work up the initiative to do things” to “neglect work”; edge weight=0.11). The 3 strongest OEI nodes (Figure 3) were D4 (“I felt downhearted and blue”; OEI=1.53), S3 (“I felt that I was using a lot of nervous energy”; OEI=1.36), and S5 (“I found it difficult to relax”; OEI=1.24). The 3 strongest IEI nodes (Figure 3) were A2 (excessive use; IEI=2.70), A1 (salience; IEI=2.08), and S6 (“I was intolerant of anything that kept me from getting on with what I was doing”; IEI=1.31). Finally, the 3 strongest BEI nodes (Figure 4) were D4 (“I felt downhearted and blue”; BEI=0.15), D6 (“I felt I wasn’t worth much as a person”; BEI=0.14), and D7 (“I felt that life was meaningless”; BEI=0.11).

In the T2-to-T3 network (Figure 2), the strongest cross-lagged edge within IA symptoms was from A5 to A2 (“lack of control” to “excessive use”; edge weight=0.11), while within psychological distress symptoms, it was from N6 to N5 (“I was aware of the action of my heart in the absence of physical exertion” to “I felt I was close to panic”; edge weight=0.17). Regarding the longitudinal effects between IA and psychological distress, the most significant cross-lagged edges were from D2 to A1 (“I found it difficult to work up the initiative to do things” to “salience”; edge weight=0.28) and from S6 to A1 (“I found it difficult to work up the initiative to do things” to “salience”; edge weight=0.13). The 3 strongest OEI nodes (Figure 3) were S6 (“I was intolerant of anything that kept me from getting on with what I was doing”; OEI=4.18), D2 (“I found it difficult to work up the initiative to do things”; OEI=1.78), and N6 (“I was aware of the action of my heart in the absence of physical exertion”; OEI=1.15). The 3 strongest IEI nodes (Figure 3) were A1 (salience; IEI=3.30), A2 (excessive use; IEI=1.65), and A5 (lack of control; IEI=1.49). Lastly, the 3 strongest BEI nodes (Figure 4) were S6 (“I was intolerant of anything that kept me from getting on with what I was doing”; BEI=0.56), S4 (“I felt I wasn’t worth much as a person”; BEI=0.15), and D7 (“I felt that life was meaningless”; BEI=0.14).

In the T1-to-T3 network (Figure 2), the strongest cross-lagged edge within IA symptoms was from A5 to A3 (neglect work; edge weight=0.07). Among psychological distress symptoms, the strongest edge was from S4 to S6 (“I found myself getting agitated” to “I was intolerant of anything that kept me from getting on with what I was doing”; edge weight=0.14). The most significant cross-lagged edges between IA and psychological distress were from D4 to A2 (“I felt downhearted and blue” to “excessive use”; edge weight=0.22) and from D4 to A1 (“I felt downhearted and blue” to “salience”; edge weight=0.21). The 3 strongest OEI nodes (Figure 3) were D4 (“I felt downhearted and blue”; OEI=3.32), S4 (“I found myself getting agitated”; OEI=1.78), and N5 (“I felt I was close to panic”; OEI=1.52). The 3 strongest IEI nodes (Figure 3) were A1 (salience; IEI=3.26), A2 (excessive use; IEI=2.37), and A5 (lack of control; IEI=0.86). The 3 strongest BEI nodes (Figure 4) were N5 (“I felt I was close to panic”; BEI=0.20), S3 (“I felt that I was using a lot of nervous energy”; BEI=0.20), and D4 (“I felt downhearted and blue”; BEI=0.19). The results of the bootstrapped difference tests for edges, OEI, IEI, and BEI across the 3 CLPNs are presented in Figure S3 in Multimedia Appendix 1.

### Accuracy, Stability Estimation, and Power Analysis

The 95% bootstrapping CIs around the edge weights for the 3 IA networks and the CLPNs were found to be small to moderate (Figures S4 and S5 in Multimedia Appendix 1), indicating that the stability of the networks is acceptable. Additionally, the analysis of network accuracy revealed a moderate level of accuracy (Figure S6 in Multimedia Appendix 1). The results of the power analysis are included in Figure S7 in Multimedia Appendix 1. When n=1200 (48.1%), the effect of correlation, sensitivity, and specificity of the networks were all acceptable (>0.6).

## Discussion

### Principal Findings

This study investigated the development of IA symptoms and the relationship between IA symptoms and psychological distress over time by applying network analysis and the CLPN approach. Central IA symptoms, such as excessive use, salience, and neglect work, were identified, and their dynamic changes within the networks were illustrated. Results of CLPN analysis revealed that edges with the highest weights were mainly from depressive symptoms to IA symptoms across all 3 waves. Depressive symptoms and stress symptoms emerged as high-OEI symptoms, while IA-related nodes were mostly high-IEI symptoms. Furthermore, depressive symptoms, such as sadness, were found to serve as bridge symptoms. These findings illuminate the intricate connections between IA symptoms and mental health symptoms, suggesting that IA may be influenced by psychological distress, especially depressive symptoms, thus shedding light on the fundamental structure underlying these relationships.

Regarding the development of IA networks, the central symptoms and network invariance were highly similar across the 2 cross-sectional networks. Symptoms with the highest EI, such as A1 (excessive use), A2 (salience), and A3 (neglect work), did not change with time. This provided evidence that continued excessive internet use, as a core symptom of problematic internet use, is closely related to other problematic internet use symptoms and is more likely to trigger other types of problematic internet use [41]. The stable network invariance of IA also suggested that specific internet addiction behaviors may not easily change, which is consistent with previous studies on a large sample of Hong Kong secondary school students. Yu et al [42] also found that the addiction problem is relatively stable as opposed to wearing off over 3 years.

However, we also found that the global strength of the T2 network was significantly higher than that of T1. Such a pattern may mirror trends observed in other behavioral addictions (eg, gambling, drug abuse), where behaviors typically do not decline or may even increase as individuals become more entrenched in their addiction [43]. Similarities between IA and other behavioral addictions have been widely highlighted, particularly regarding characteristics including excessive use, tolerance, and withdrawal symptoms. These parallels have led to calls for recognizing IA as a distinct disorder [34]. Another possible explanation for the higher global strength of the T2 network may be the combined effects of the pandemic outbreak and academic pressure. Previous studies have noted an increase in IA addiction severity within the general population in China following the pandemic [44]. Cross-national studies have also reported a higher prevalence of IA among college students during this period [45,46]. Moreover, by their third year, college students are typically more engaged in major coursework and face challenges, such as securing internships, which may restrict their internet use. Academic pressures may also diminish the appeal of internet use among students. These hypotheses warrant further exploration through in-depth investigations, such as integrating latent profile analysis with network analysis for subgroup-specific investigations of symptom evolution patterns among populations with heterogeneous developmental trajectories.

Nodes related to psychological distress emerged as central predictive symptoms, while IA symptoms were primarily identified as symptoms being predicted. For instance, nodes D4 (“I felt downhearted and blue”), S4 (“I found myself getting agitated”), and D2 (“I found it difficult to work up the initiative to do things”) exhibited high OEI, whereas nodes A2 (salience) and A1 (excessive use) were identified as high-IEI symptoms. This could be because excessive internet use is often studied as a coping strategy aimed at alleviating uncomfortable emotions, such as loneliness, depression, and social anxiety [47]. According to the I-PACE model and compensatory internet use theory, using the internet to avoid negative emotions can help regulate emotions temporarily, thereby improving negative moods and providing a sense of personal and social competence [17]. When individuals feel emotionally overwhelmed, anxious, stressed, or depressed, they may have reduced capacity for conscious coping and resilience, leading them to use the internet as an escape from reality [48]. Consequently, they may become trapped in self-destructive, addictive behaviors due to a limited ability to cope with depression, anxiety, and stress [49]. Adolescence is a critical period for developing emotional and cognitive capacities and forming lasting connections but also carries a heightened risk for behavioral addictions, such as IA, as well as gambling and eating disorders [50]. Therefore, it is essential to recognize adolescents’ vulnerability to digital threats, especially IA and related mental disorders, such as depression [51,52].

However, recent studies investigating the directionality of associations between psychological distress and IA have found mixed results. Several longitudinal studies have clarified reciprocal relationships between IA and depressive symptoms over time among college students [28,53]. Nevertheless, these studies were conducted at different time points compared to our data collection, specifically before the onset of the pandemic. Our findings provide a reflection of the directional relationship between IA and mental health during the occurrence of a major public health event. Bidirectional influences might emerge in adolescents with severe IA symptoms meeting diagnostic criteria or in clinical samples as well [54,55]. Thus, additional studies are needed to further clarify the predictive effects between psychological distress and IA across different population samples, such as clinical samples and samples of other ages. Moreover, several studies have diverged from our findings and found that IA and problematic internet game use symptoms exhibit high OEI among early adolescents, suggesting that IA symptoms could initiate other symptoms [6,55,56]. Such discrepancy may stem from differences in the study samples, with the earlier research examining elementary students in China, while ours involved college students who may experience distinct stressors that affect their responses to IA. This distinction highlights the importance of considering stress levels and types across age groups. To better conclude this point, we considered different forms of distress and aimed to more broadly assess the negative emotional experiences that college students might encounter, rather than focusing solely on one specific type. Through this way, we sought to explore which of the common emotional states—depression, anxiety, or stress—is more strongly associated with problematic internet use among college students.

This study also found that across all 3 CLPNs, the highest-weighted edges originated from the “depressive symptoms” dimension to IA. Consistent with prior research, depressive symptoms demonstrated a stronger association with IA than stress or anxiety [12,30]. This pattern is further supported by studies on specific types of internet use. Longitudinal research examining both cross-lagged and autoregressive effects between depression and internet gaming disorder (IGD) has consistently shown that depression predicts IGD in children and adolescents, whereas the reverse effect has not been observed [57,58]. Similarly, a previous longitudinal study on depression and smartphone addiction in Chinese adolescents identified a 1-way predictive effect of depression on smartphone addiction, with no reverse effect [59]. These findings imply that depression may be a predictor of IA symptoms [60,61]. A possible explanation is that during adolescence, the ability to regulate emotions is limited, resulting in adolescents being more prone to mental health problems related to IA, and depression is among the most common diseases [62,63]. A recent government report highlighted depression as a key mental health concern among adolescents requiring intervention, while also drawing attention to the rising issue of behavioral addictions linked to internet use in Chinese youth [64]. Building on existing research, depression is often linked to social withdrawal, diminished motivation for real-world activities, and a greater dependence on online platforms for emotional regulation or escapism, all of which may heighten the risk of IA [65]. One study also found that both depression and IA are linked to a short allele in the promoter region of the serotonin transporter gene (*SS-5HTTLPR*), suggesting that individuals with both conditions may share similar genetic and personality traits [66]. Our findings align with those of a meta-analysis that examined the relationship between IA and depression across a sample of individuals aged 13-64 years [14,67]. The results similarly indicated that people with depression tend to engage in more intense internet use. In contrast, anxiety and stress, although also significant, may manifest differently in online behaviors, such as seeking reassurance or information rather than prolonged engagement [68].

Moreover, our findings revealed that depressive symptoms, particularly D4 (“I felt downhearted and blue”), served as relatively stable bridge symptoms with high strength across all 3 networks. These bridge symptoms represent priority treatment targets, as their deactivation might prevent the emergence of other co-occurring symptoms [69]. This suggests an understanding of how co-occurrence is maintained, with depressive symptoms playing a pivotal role. Past research using network analysis has similarly indicated that a *sad mood* acts as a bridging symptom, driving young individuals toward *mood modification* through short-video platforms [70]. Another network analysis focusing on the co-occurrence of IA and residual depressive symptoms among adolescents identified that suicide ideation is one of the bridge symptoms across various mental disorders [71]. One possible reason for this phenomenon is that the prevalence of depressive symptoms in China is comparatively higher than the worldwide average. Meta-analytic reviews indicated that the prevalence of depressive symptoms among Chinese adolescents rose from 24.3% before the pandemic [72] to 28.3% [73] during the pandemic. Meanwhile, depression is highly comorbid with behavioral problems, such as substance abuse [74]. However, individuals suffering from depressive symptoms often seek recreational activities, including internet use, as a means of stress relief. Internet use, particularly video gaming, is commonly regarded as an emotion-focused coping strategy [75]. Consequently, individuals experiencing depressive symptoms may turn to excessive internet use as a compensatory mechanism to mitigate their negative effect.

### Implications

Our research not only enhances the theoretical framework of the problematic internet use model [76] but also emphasizes the importance of identifying central and bridge symptoms in addressing addiction-related issues. By expanding this model, we elucidated which psychological distress factors contribute to the engagement in addictive behaviors. These findings offer valuable insights into the development of effective prevention and intervention strategies for IA. Specifically, greater attention should be given to youth experiencing psychological distress, particularly depressive symptoms, as they may turn to the internet as a coping mechanism or avoidance strategy. It is also important to address the connection between bridge symptoms to prevent the escalation of their links to other mental health issues. When working with adolescents exhibiting problematic internet use, incorporating an assessment of concurrent distress—especially depressive symptoms—into the treatment process is crucial and can be a cost-effective approach.

### Limitations and Further Research

This study has several limitations. First, it relied on self-reported measures of IA and psychological distress, which may have subjective biases, such as the influence of social desirability and recall bias. Adolescents often overestimate their internet activity as well, making it challenging to obtain an objective assessment through self-reported instruments [77]. Future studies could address this issue by integrating self-reported data with objective measures, such as internet usage logs or other behavioral tracking methods, to reduce potential biases. Additionally, incorporating alternative clinical assessment tools and scales could further enhance measurement accuracy. Second, the sample was drawn from a single college in China, which limits the generalizability of the findings to other populations with varying ages and experiences. However, it is worth noting that our participants represented most provincial administrative regions of China. Moreover, the IAT-20 scale used in this study does not distinguish between different types of internet use, such as social media, web browsing, online shopping, or gaming. Future research should explore these specific subdomains and examine their distinct relationships with mental health. Finally, although our study highlighted the impact of psychological distress, particularly depression, on IA symptoms at follow-up, longitudinal studies with extended durations and multiple time waves are needed to explore common vulnerabilities and potential mechanisms in greater depth, thereby enhancing our understanding of this field.

### Conclusion

This study explored the evolution of IA and the longitudinal relationship between IA symptoms and psychological distress among Chinese youth, using network analysis and CLPNs. The findings reveal stable central symptoms of IA—specifically, excessive use (A2), salience (A1), and lack of control (A5)—among college students. Importantly, the results indicate that psychological distress predicts IA symptoms rather than the reverse. Among the various forms of distress, depressive symptoms, such as “I felt downhearted and blue” (D4) and “I found it difficult to work up the initiative to do things” (D2), exhibit the highest predictive effect. Furthermore, depressive symptoms may serve as bridge symptoms, linking the co-occurrence of these conditions. These results provide evidence for understanding the directional relationship between the central characteristics of distress symptoms and IA. Psychological distress, especially depressive symptoms, may play a central role in activating IA symptoms over time. The study underscores the importance of depressive symptoms in their co-occurrence with IA, indicating that the key and bridge symptoms identified in this study can be prioritized as targets for preventing and treating IA in Chinese youth. Through identification and early intervention of depressive symptoms, we may avoid the progression of co-occurring issues, leading to more effective treatment outcomes.

## Appendix

Multimedia Appendix 1 Internal consistencies of 6 factors of the Internet Addiction Test (IAT; Table S1); confirmatory factor analysis of IAT-20 (Table S2); bootstrapped difference test of edges and expected influence for internet addiction (IA) networks (Figure S1); cross-lagged panel networks (CLPNs), including all cross-lagged edges (Figure S2); bootstrapped difference test of edges, out–expected influence, in–expected influence, and bridge–expected influence for CLPNs (Figure S3); bootstrapped 95% CIs around each edge weight for IA networks (Figure S4); bootstrapped 95% CIs around each edge weight for CLPNs (Figure S5); stability of centrality and edge measures in IA networks and CLPNs (Figure S6); power analysis simulation results of IA networks (Figure S7).

## Abbreviations

BEI: bridge–expected influence
CLPN: cross-lagged panel network
CS: centrality stability
DASS: Depression Anxiety Stress Scale
EI: expected influence
IA: internet addiction
IAT: Internet Addiction Test
IEI: in–expected influence
IGD: internet gaming disorder
I-PACE: interaction of person-affect-cognition-execution
OEI: out–expected influence

## Data Availability

The data that support the findings of the study are available upon request from the corresponding author.
