# Supplementary material for: The Longitudinal Effect of Psychological Distress on Internet Addiction Symptoms Among Chinese College Students: Cross-Lagged Panel Network Analysis
Source: J Med Internet Res. 2025 May 2;27:e70680. doi: 10.2196/70680 (PMC12084773; doi:10.2196/70680)
Supplement: Multimedia Appendix 1 [file jmir_v27i1e70680_app1.doc]

**Multimedia Appendix 1**

**The longitudinal effect of psychological distress on internet addition symptoms among Chinese college students: a cross-lagged panel network analysis**

Table S1. The internal consistencies of six factors of IAT.

Table S2. Confirmatory factor analysis of IAT-20.

Figure S1. Bootstrapped difference test of edges and expected influence for IA networks.

Figure S2. The cross-lagged panel networks (including all cross-lagged edges).

Figure S3. Bootstrapped difference test of edges, out-expected influence, in-expected influence and bridge expected influence for CLPN.

Figure S4. Bootstrapped 95% confidence intervals around each edge weight for IA network.

Figure S5*.* Bootstrapped 95% confidence intervals around each edge weight for CLPN.

Figure S6. Stability of centrality and edge measures in IA network and CLPN.

Figure S7. The power analysis simulation results of IA networks.

**Tables S1. Internal consistencies of six factors of IAT**

| Variable |  | internal consistencies |
| --- | --- | --- |
| salience | T1 | 0.75 |
|  | T2 | 0.80 |
|  | T3 | 0.81 |
| excessive use | T1 | 0.75 |
|  | T2 | 0.80 |
|  | T3 | 0.80 |
| neglect work | T1 | 0.63 |
|  | T2 | 0.73 |
|  | T3 | 0.74 |
| anticipation | T1 | 0.30 |
|  | T2 | 0.44 |
|  | T3 | 0.50 |
| lack of control | T1 | 0.73 |
|  | T2 | 0.78 |
|  | T3 | 0.77 |
| lack of social life | T1 | 0.34 |
|  | T2 | 0.46 |
|  | T3 | 0.48 |

**Tables S2. CFA of IAT-20**

| Variable | Model |  | *RMSEA* | *CFI* | *TLI* | *SRMR* |
| --- | --- | --- | --- | --- | --- | --- |
| Internet addiction | 6 factor model | T1 | 0.062 | 0.925 | 0.905 | 0.041 |
|  |  | T2 | 0.075 | 0.922 | 0.900 | 0.046 |
|  |  | T3 | 0.075 | 0.927 | 0.903 | 0.044 |
|  | 1 factor model | T1 | 0.060 | 0.932 | 0.921 | 0.035 |
|  |  | T2 | 0.077 | 0.931 | 0.919 | 0.038 |
|  |  | T3 | 0.077 | 0.923 | 0.910 | 0.042 |

*NOTE*: χ2 = Chi-Square Test, *df* = Degrees of Freedom, RMSEA = Root Mean Square Error of Approximation, CFI = Comparative Fit Index, TLI = Tucker-Lewis Index, SRMR = Standardized Root Mean Square Residual.

**Figure S1. Bootstrapped difference test of edges and expected influence for IA networks**


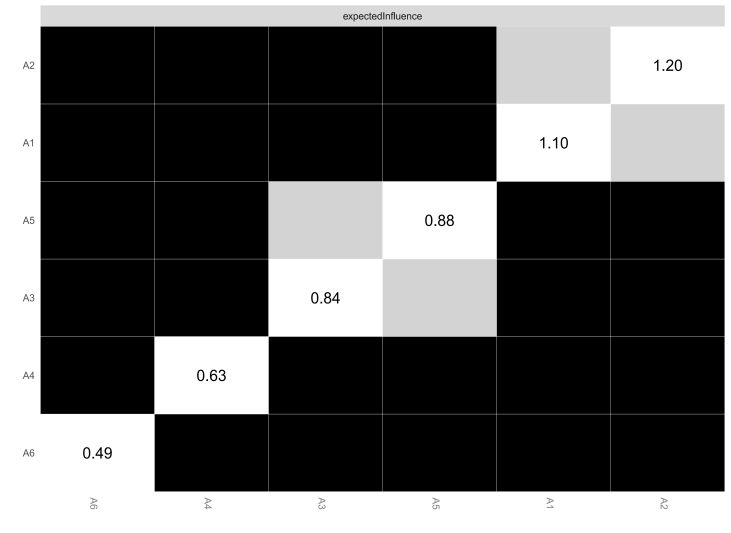

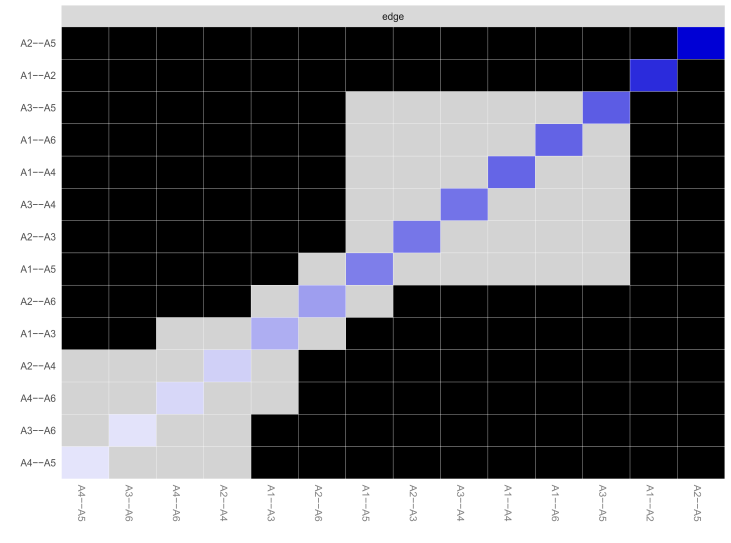

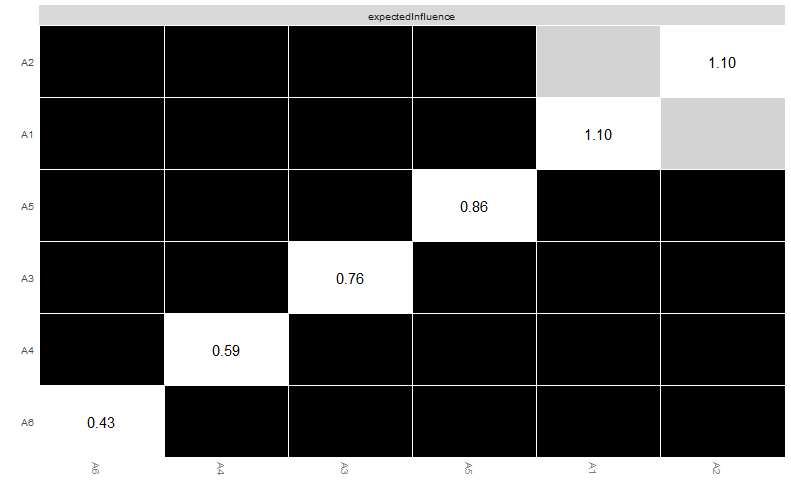

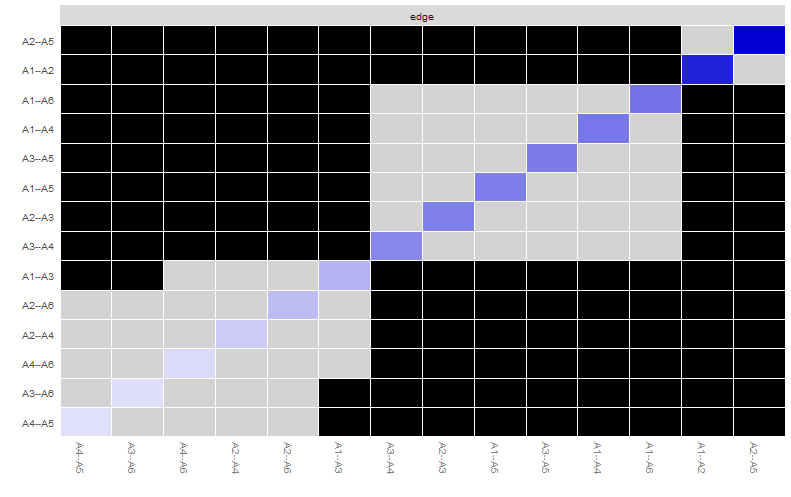


A T1

B T2


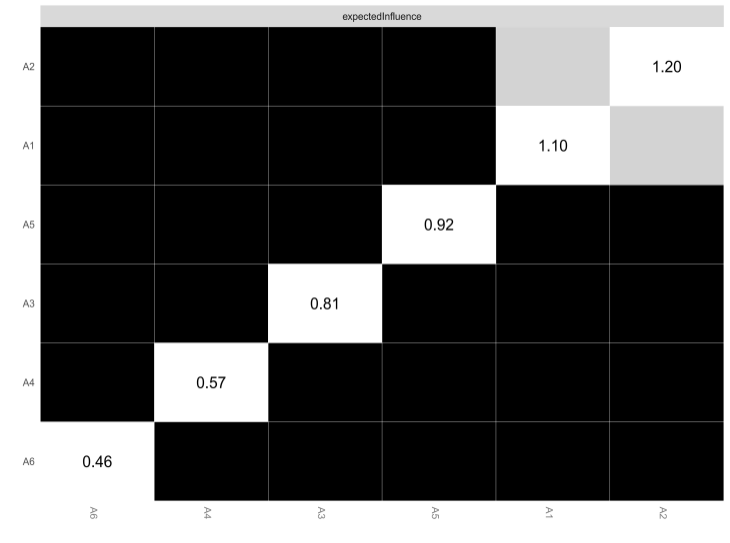

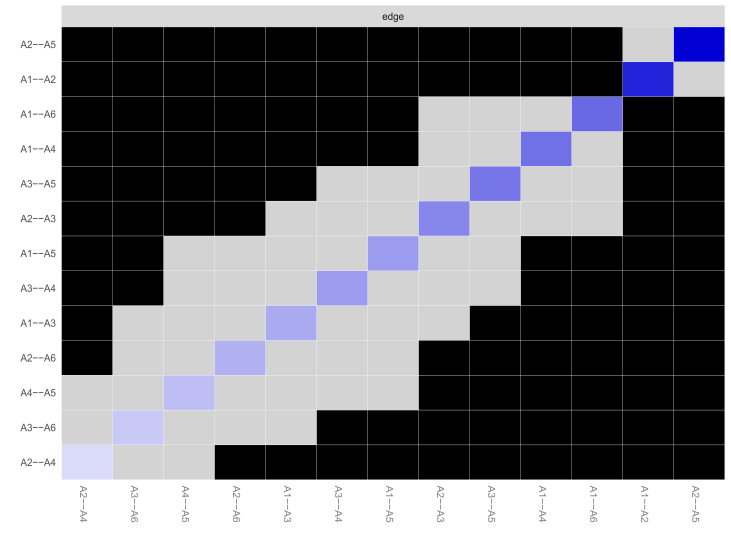


C T3

**Figure S2. The cross-lagged panel networks (including all cross-lagged edges)**


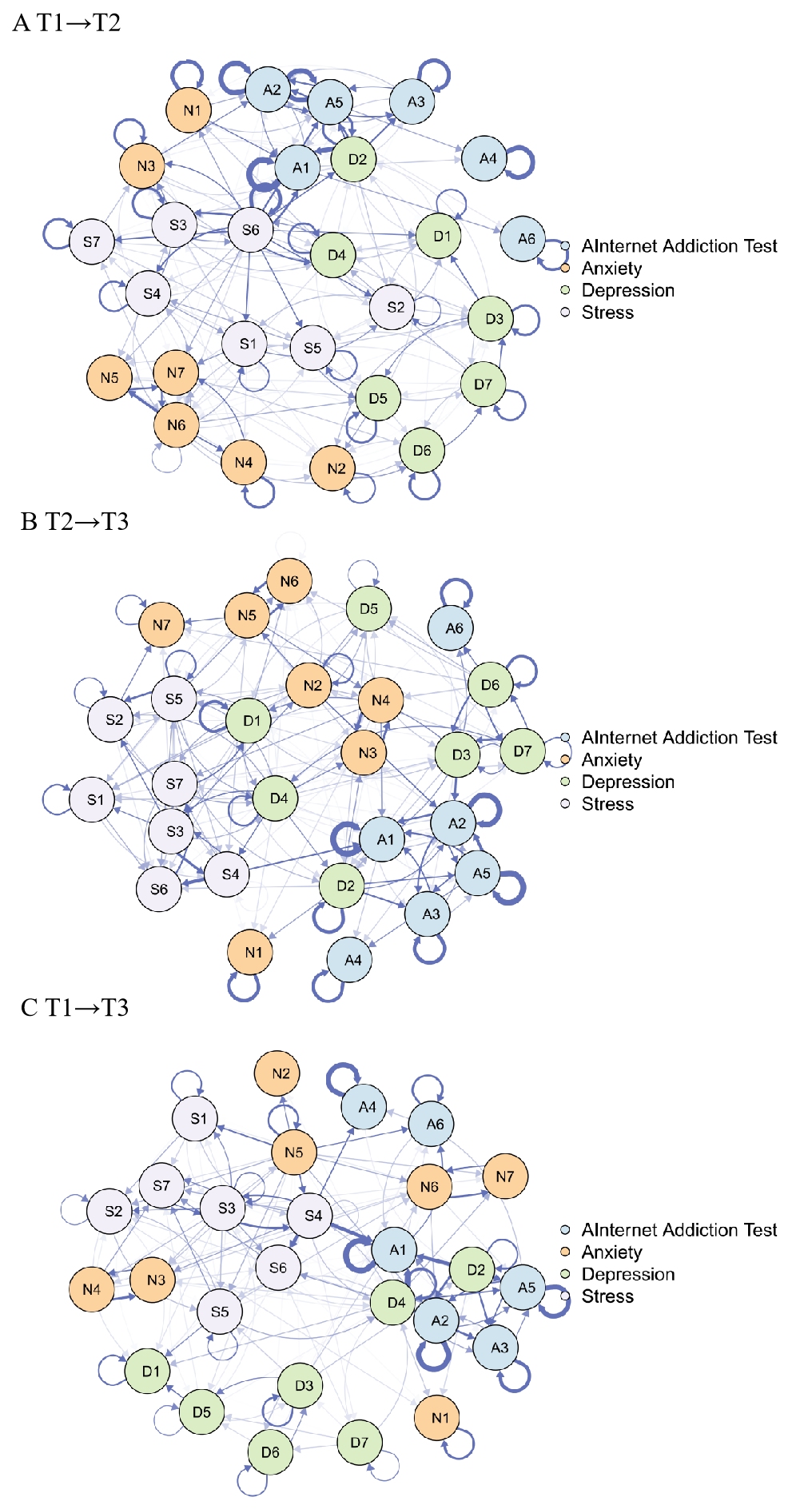


**Figure S3. Bootstrapped difference test of edges, out-expected influence, in-expected influence and bridge expected influence for CLPN**


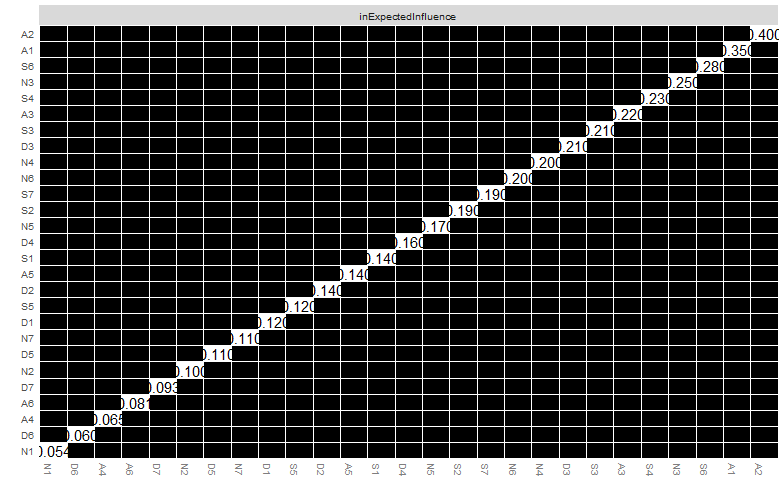

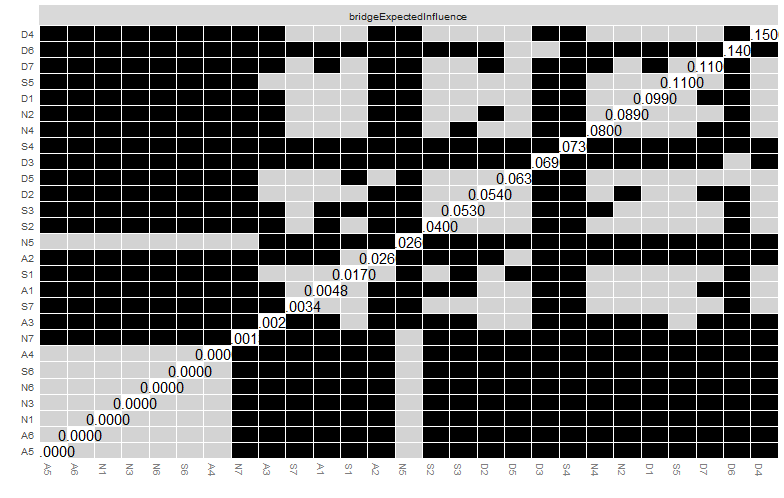

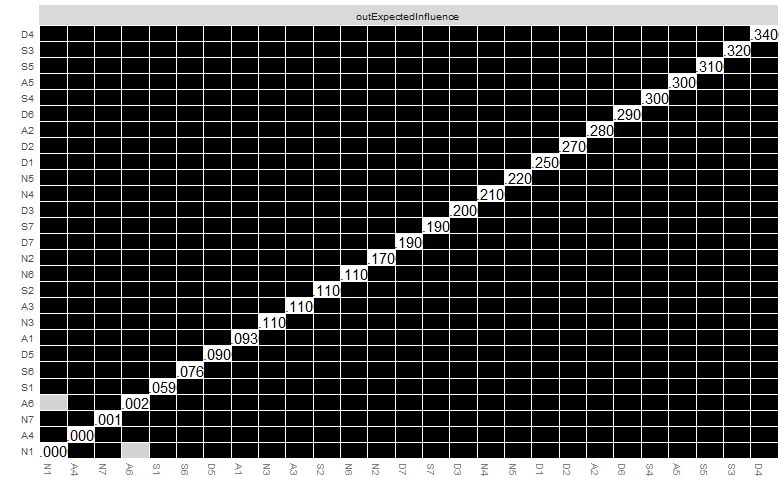

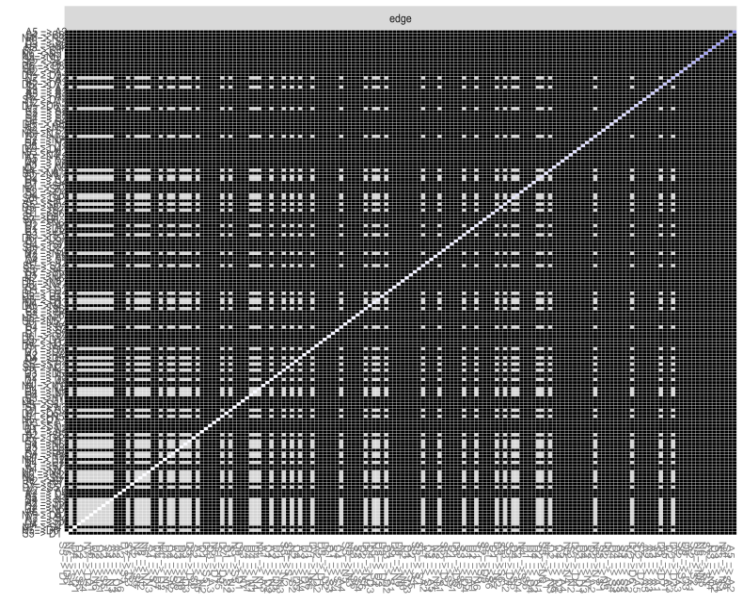


A T1→T2


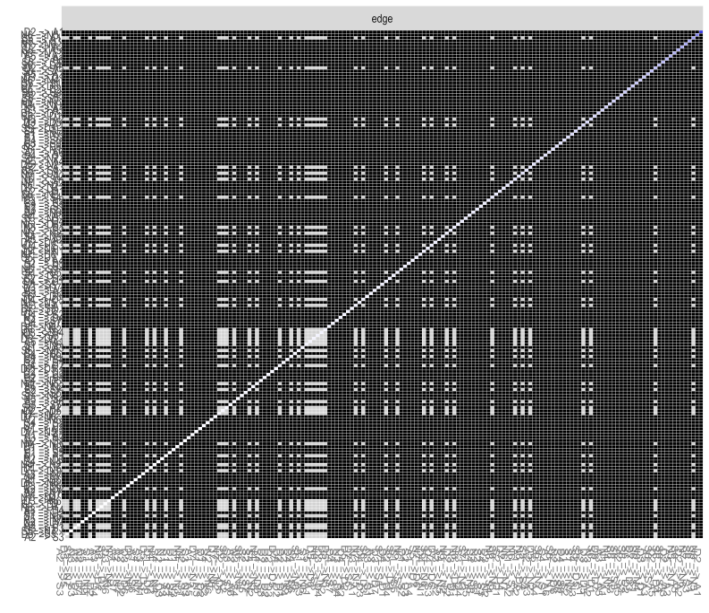

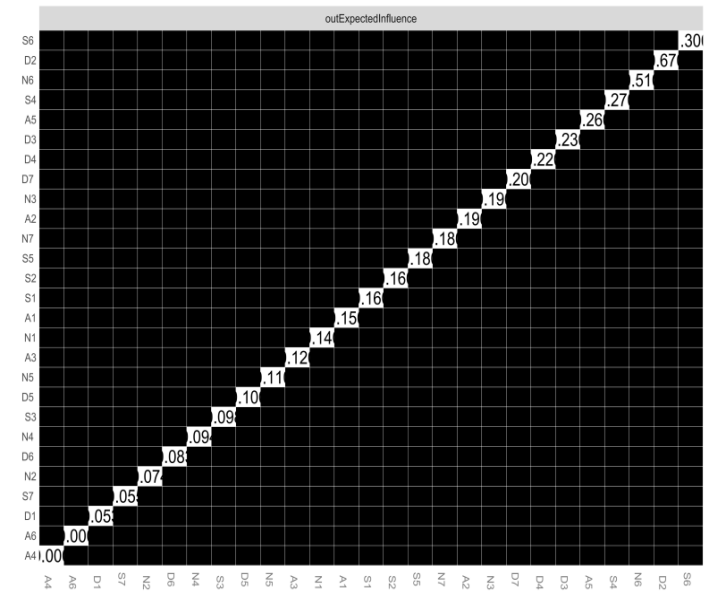

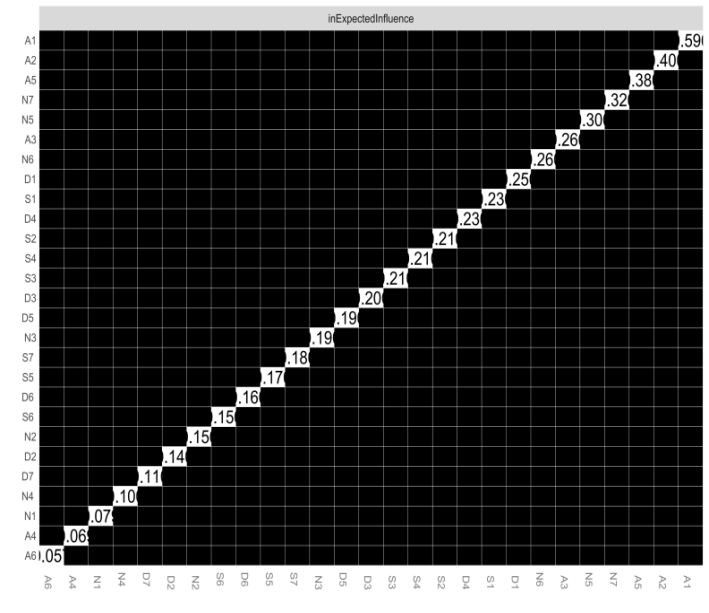

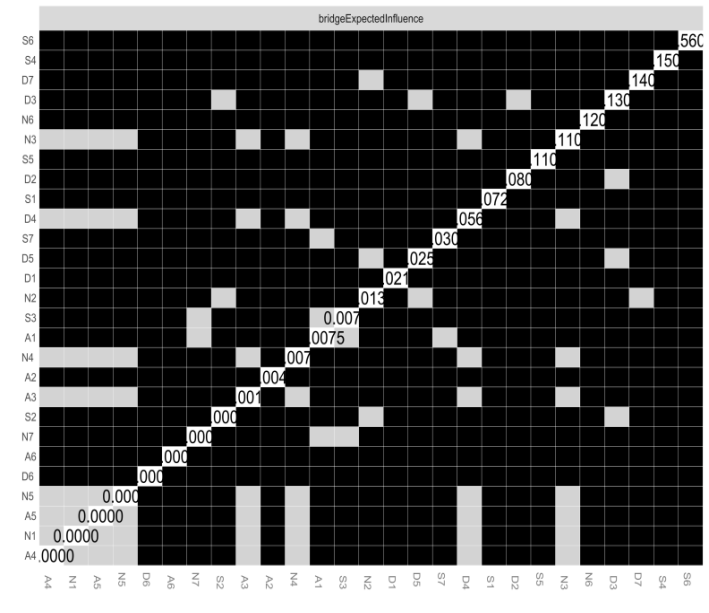


B T2→T3


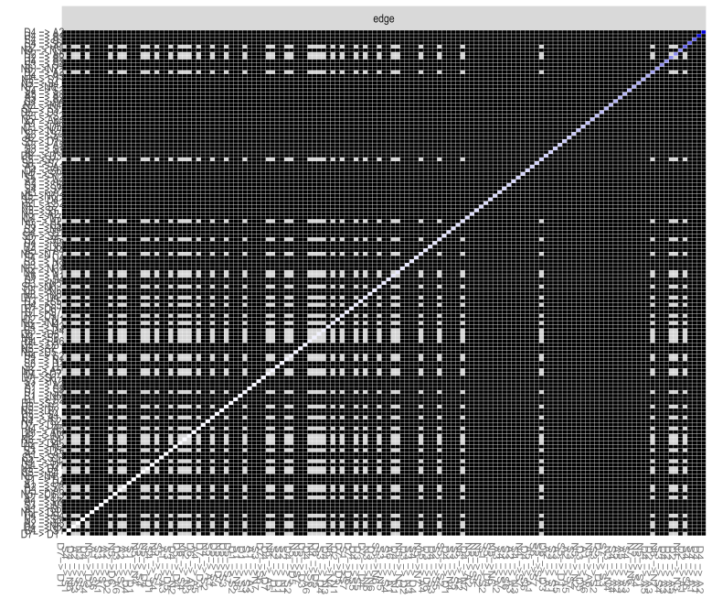

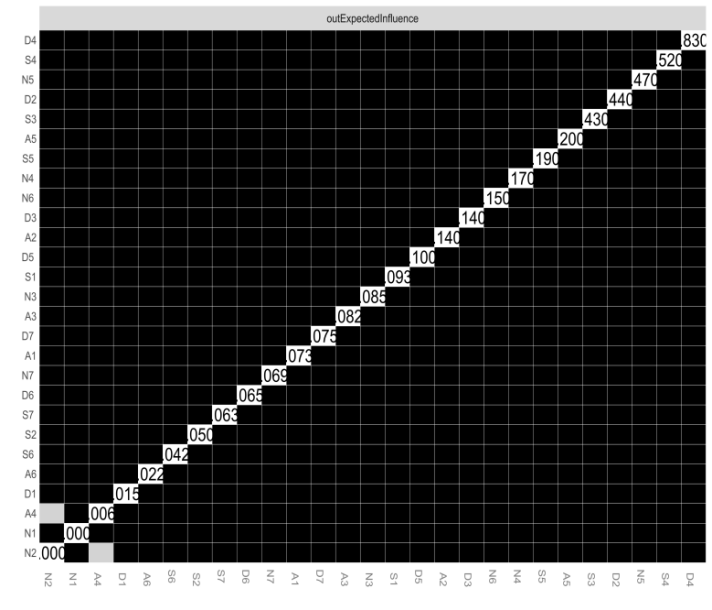

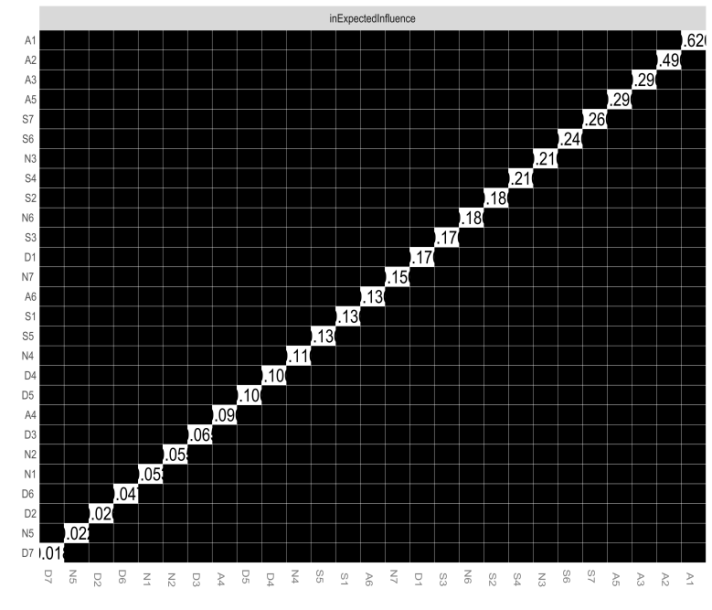

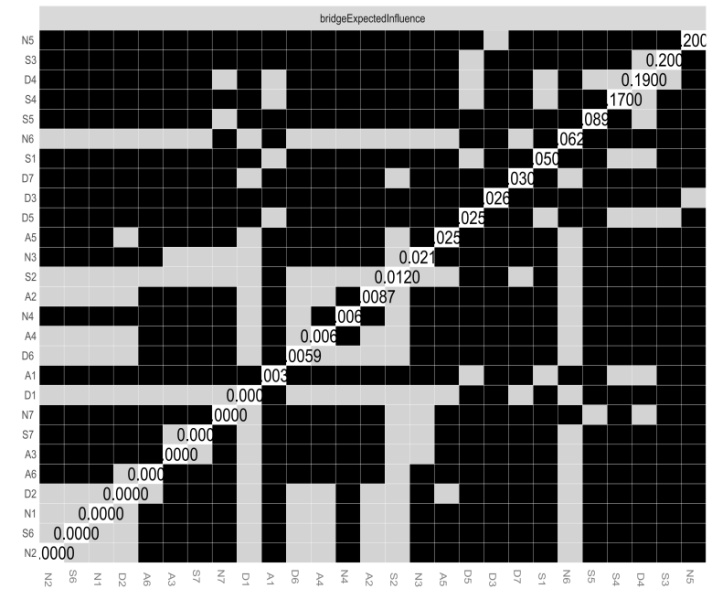


C T1→T3

**Figure S4. Bootstrapped 95% confidence intervals around each edge weight for IA network**


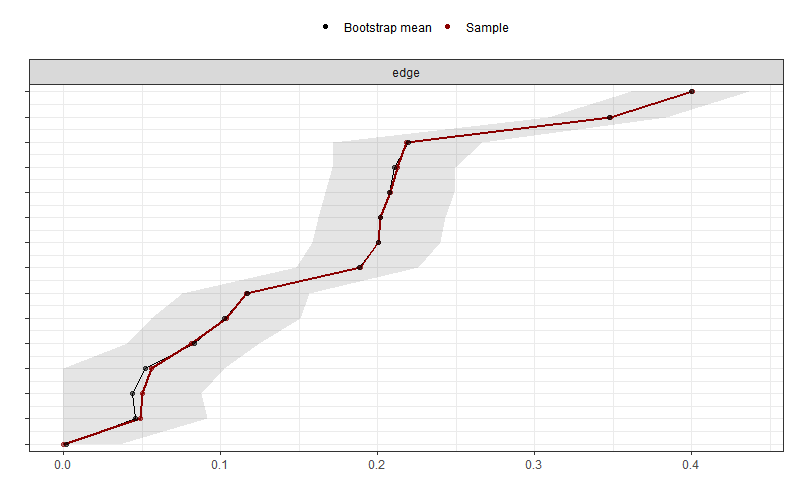

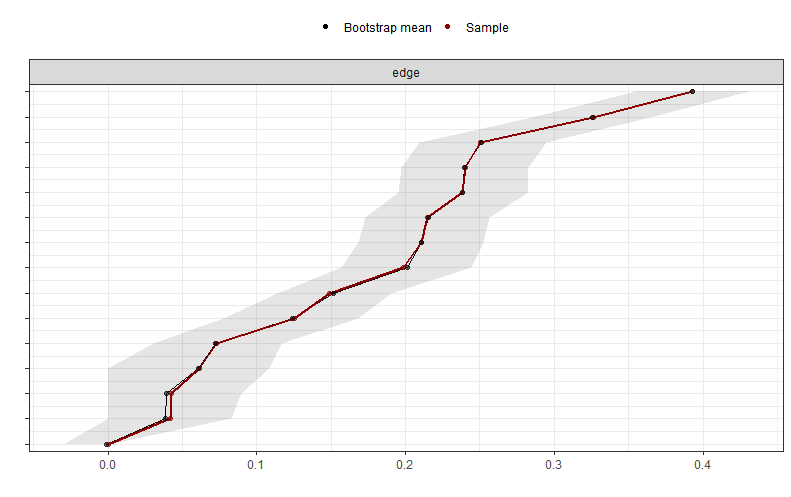

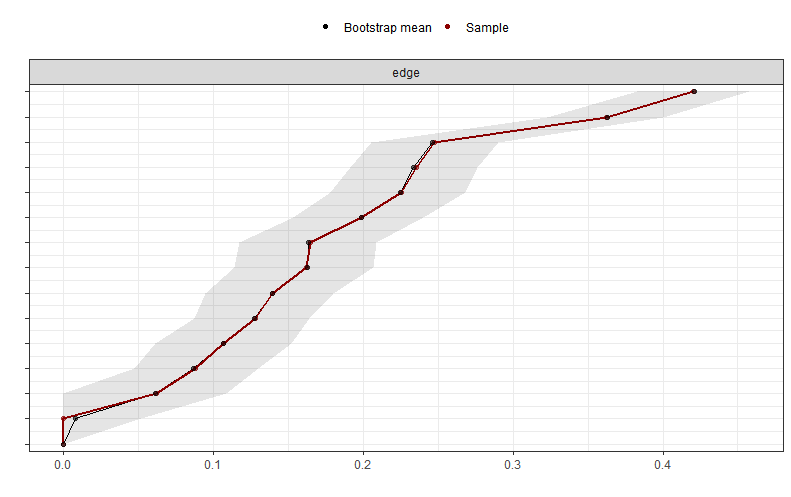


A T1

B T2

C T3

**Figure S5.** **Bootstrapped 95% confidence intervals around each edge weight for CLPN**


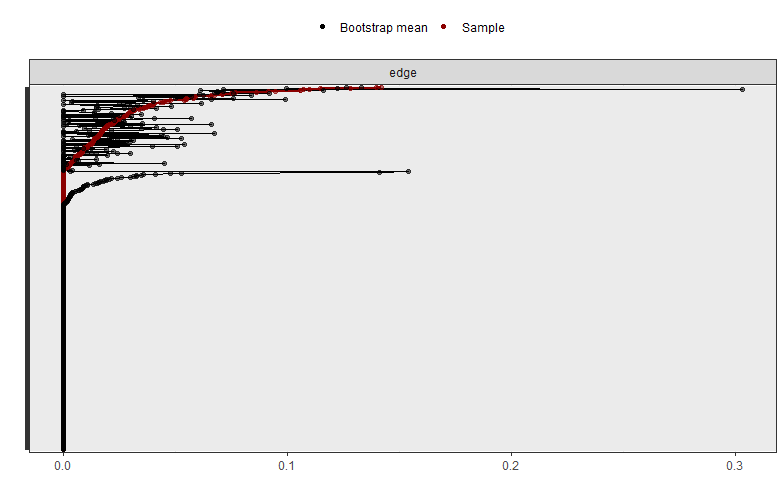

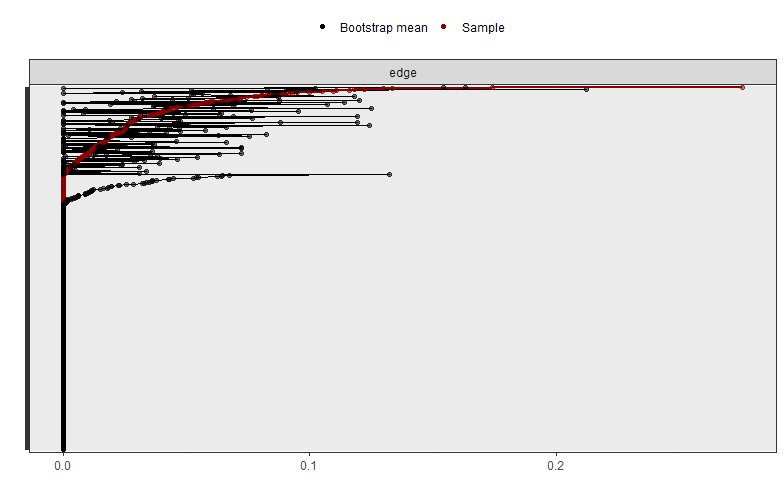

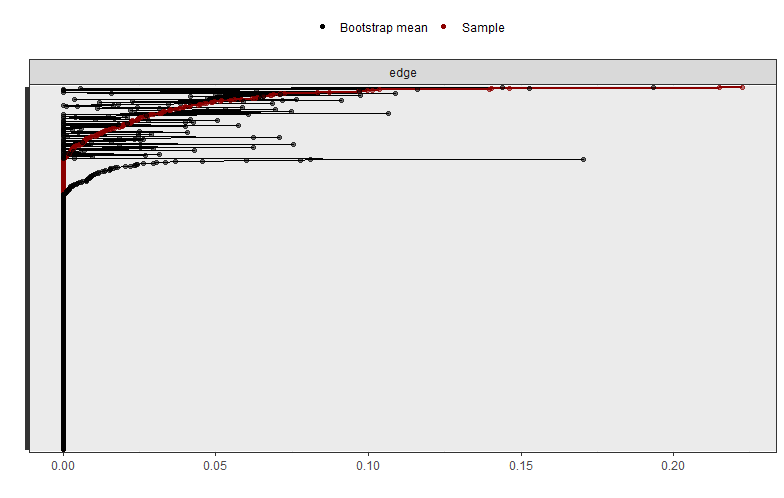


A T1

B T2

C T3

**Figure S6. Stability of centrality measures in IA network and CLPN**


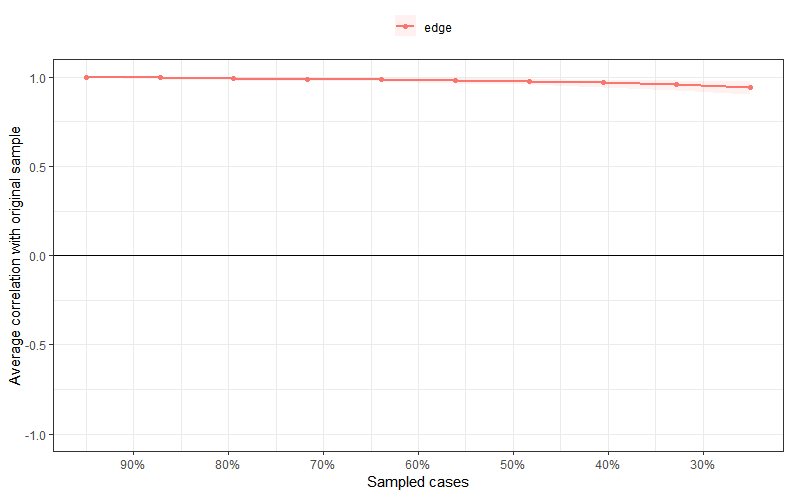

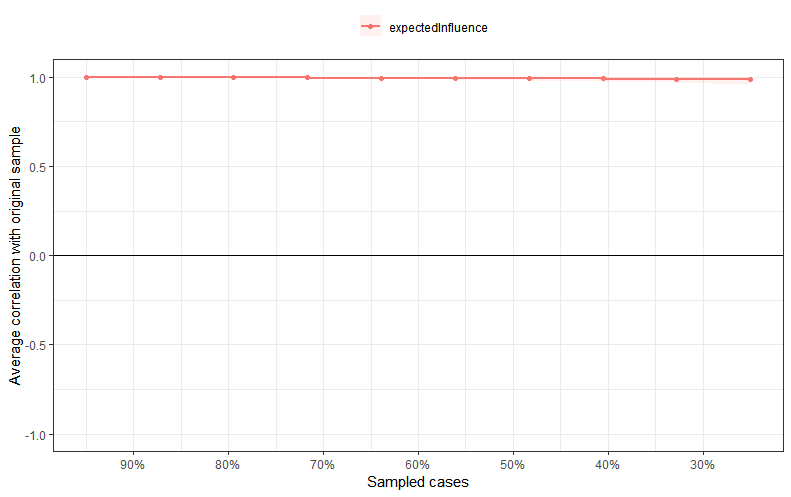


A T1


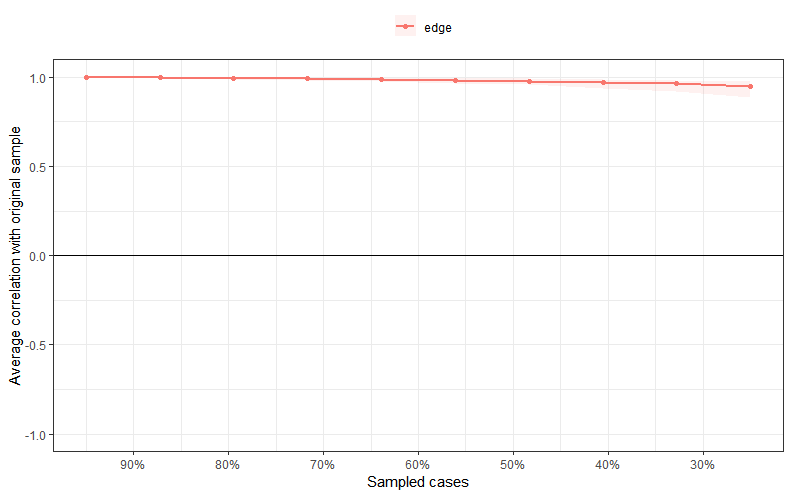

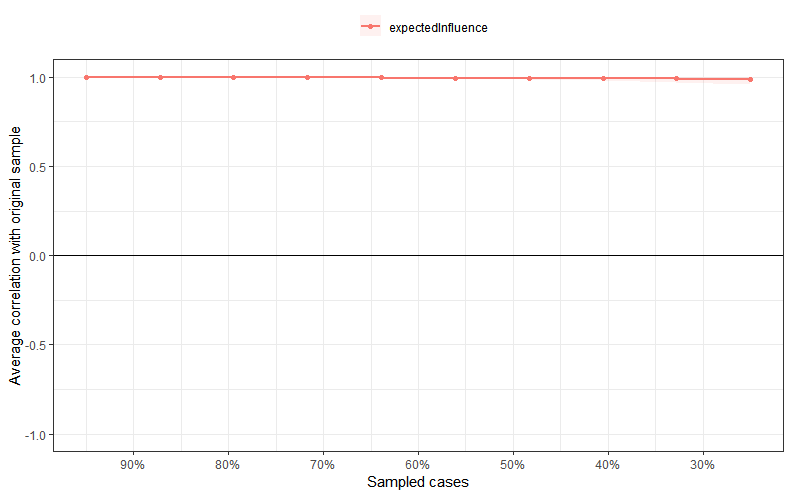


B T2


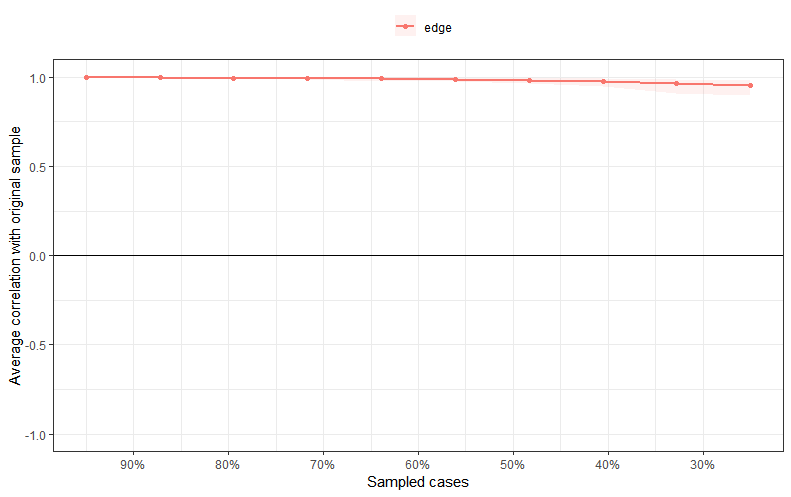

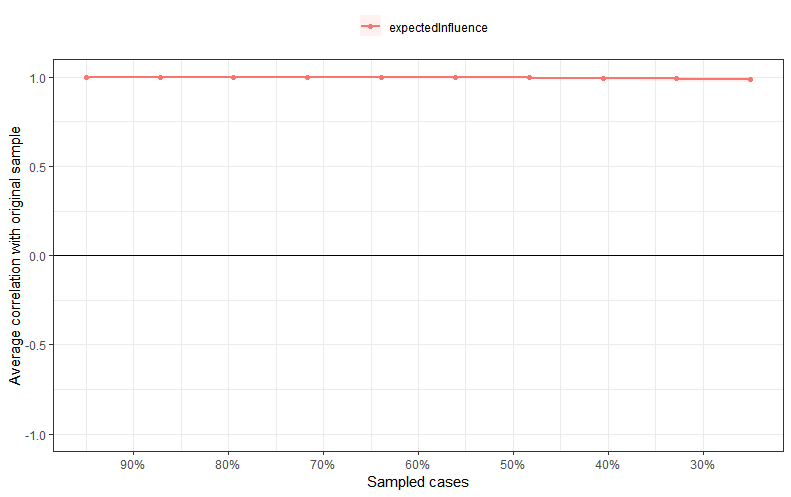


C T3


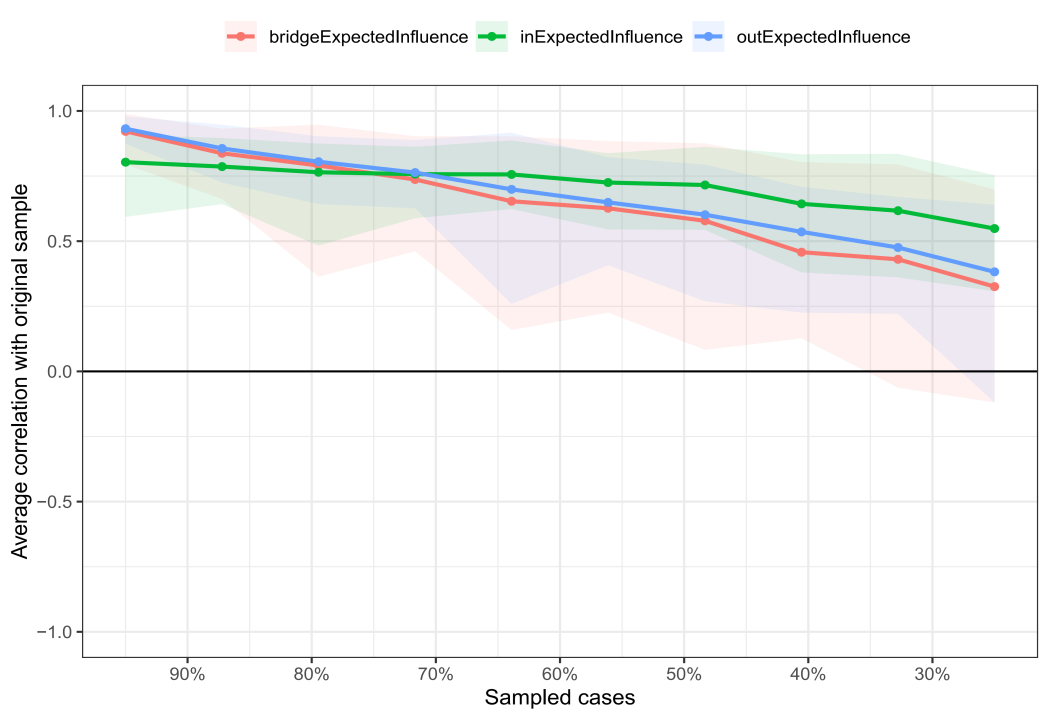


A T1→T2


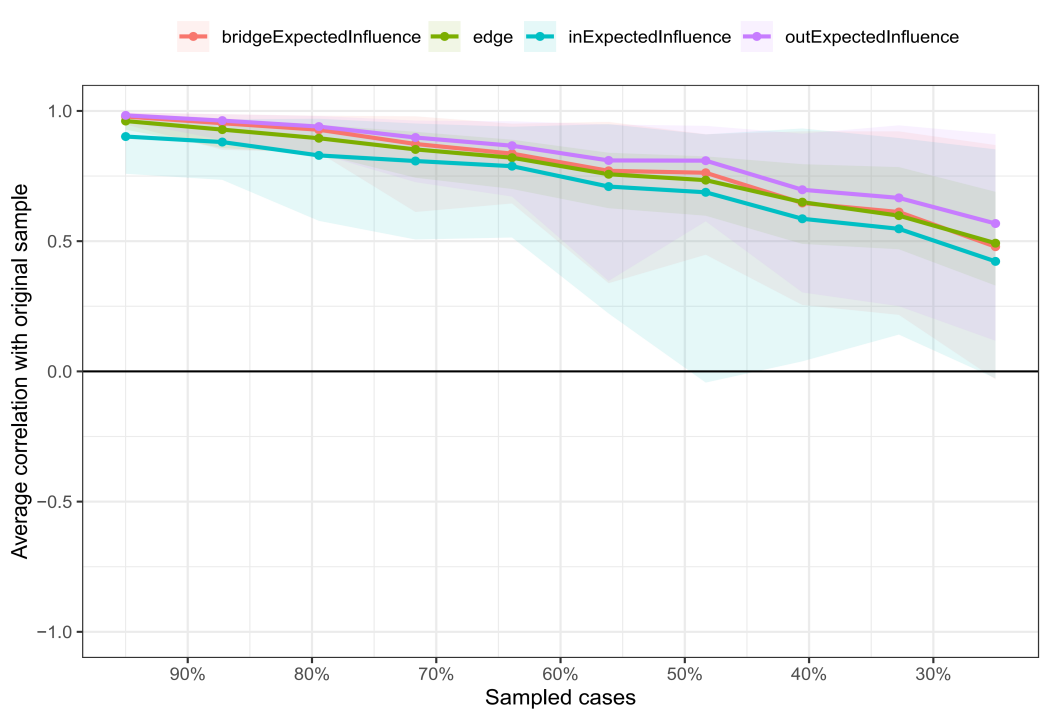


B T2→T3


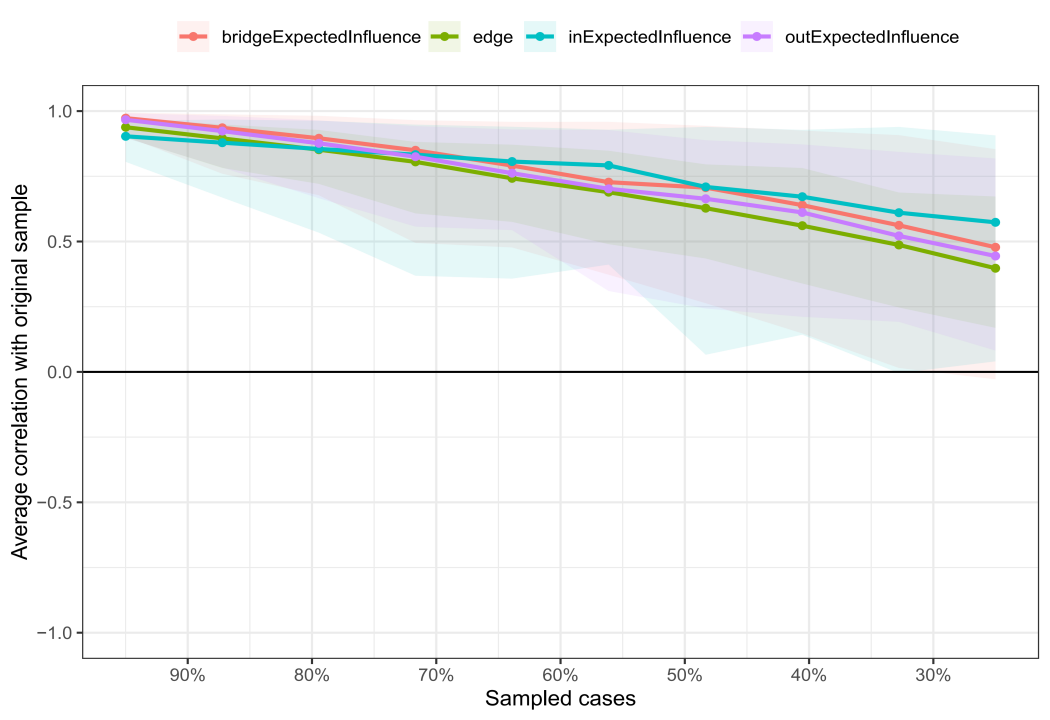


C T1→T3

**Figure S7. The power analysis simulation results of IA networks**


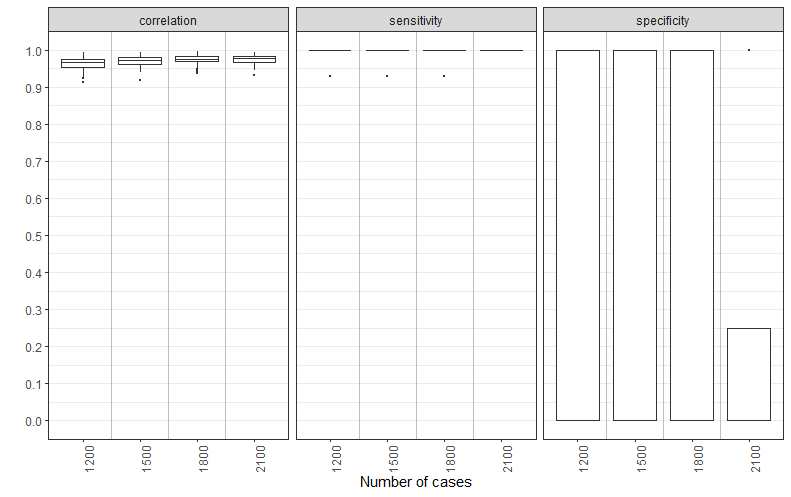

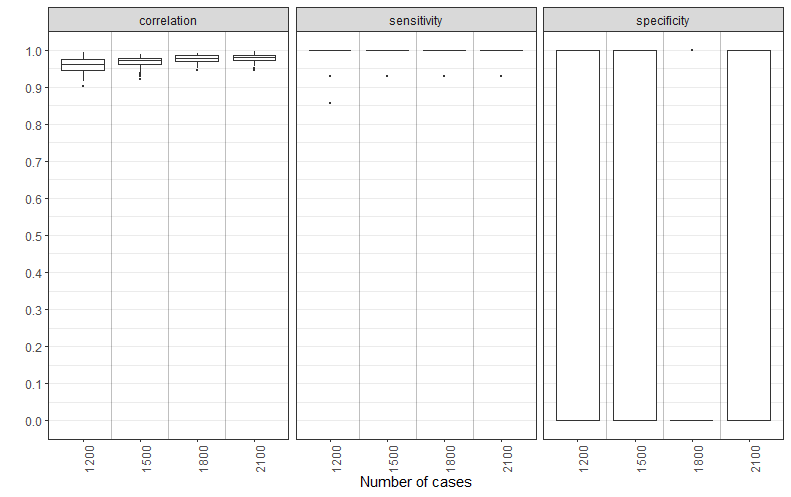

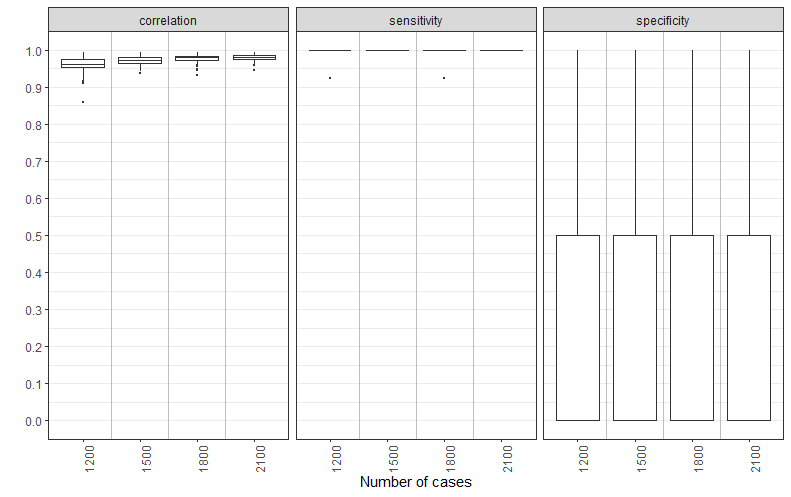


A T1

B T2

C T3
